# Studying the Presentation of Paget Disease of the Breast

**DOI:** 10.7759/cureus.41057

**Published:** 2023-06-27

**Authors:** Iqbal S Grewal, Thiru Rajagopal

**Affiliations:** 1 College of Medicine, California Northstate University College of Medicine, Elk Grove, USA; 2 General Surgery, Mercy General Hospital, Sacramento, USA

**Keywords:** paget cells, nipple ulceration, breast cancer. young adult, breast cancer, paget's disease of the breast

## Abstract

Paget disease of the breast is a rare breast cancer that accounts for 1-3% of all new presentations of breast cancer. It is characterized by an eczematous and ulcerative lesion of the nipple that may extend to the areola. Nearly 90% of cases are associated with underlying breast cancer. We report the presentation of Paget disease of the breast in a 41-year-old female, who presented due to a one-year history of an eczematous lesion of the left nipple area with no palpable mass, who was later found to have high-grade invasive ductal carcinoma treated with unilateral mastectomy and sentinel node biopsy.

## Introduction

Breast cancer accounts for 30% of all new female cancers each year [1]. With a lifetime risk of 13% and an increasing incidence rate every year, breast cancer has become a major topic of concern and discussion for further advancement of care. The American Cancer Society predicts that there will be nearly 300,000 new diagnoses of breast cancer in 2023 along with over 40,000 deaths caused by breast cancer, making it the second leading cause of death in women [2].

Breast cancer is typically viewed as being either ductal or lobular in origin and invasive or in situ in nature. However, there are a multitude of other rare presentations of breast cancer, such as Paget disease of the breast. In 1874, Sir James Paget discovered a condition involving nipple ulceration that was associated with breast cancer that would go on to be known as Paget disease of the breast (PDB) [3]. PDB is a rarer presentation of breast cancer accounting for only 1-3% of cases of breast cancer. It is associated with an underlying breast cancer in 85-88% of cases [2].

In this case report we will delve into the presentation of PDB in an otherwise healthy 41-year-old female with no family history of breast or ovarian cancer, no palpable mass, and no previous breast-related issues who presented with an eczematous lesion of the nipple. We will discuss the treatment option this patient chose along with the outcome and explore the various treatment options being considered for PDB.

## Case presentation

**Figure 1:**
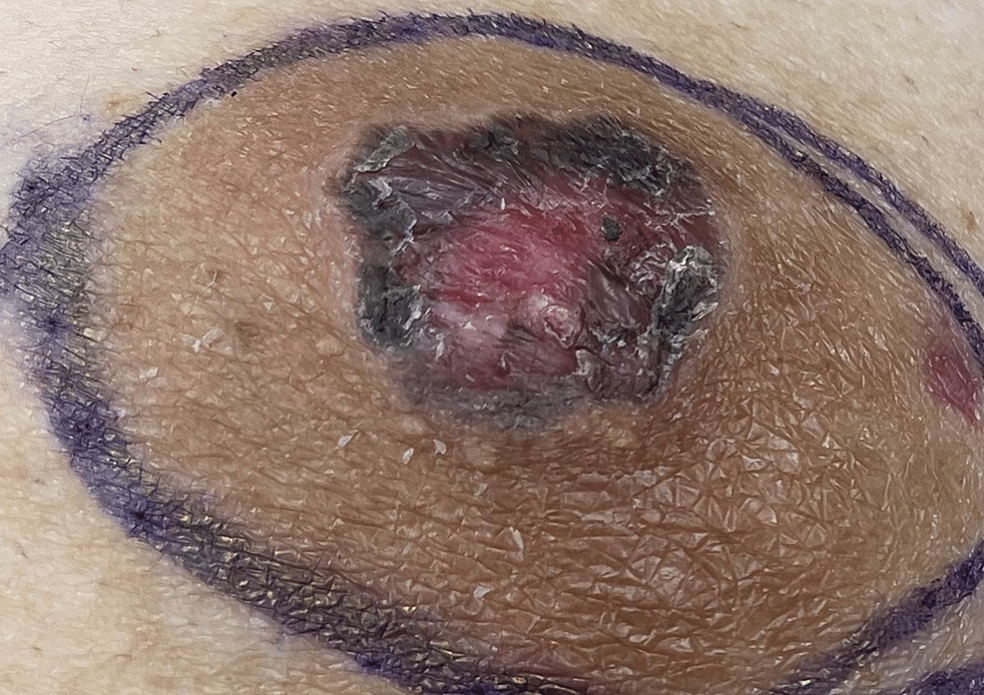
Image of ulcerated nipple lesion

**Figure 2:**
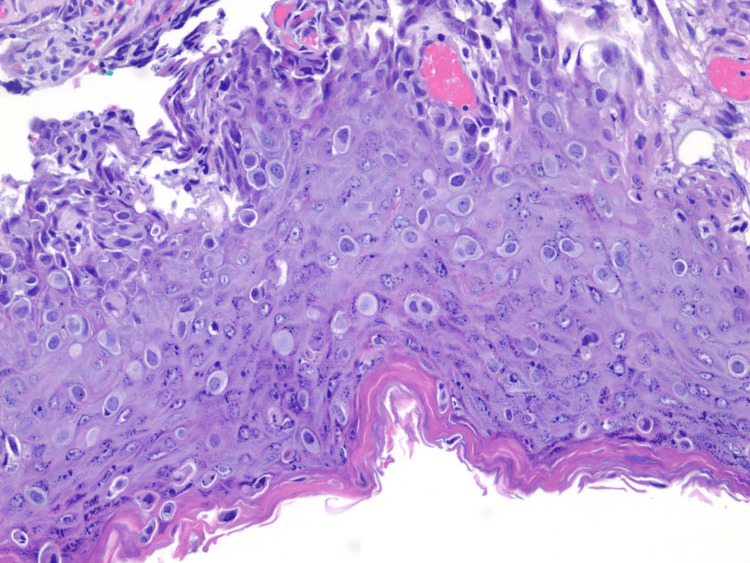
Pathology slide displaying Paget cells

A 41-year-old female presented with the primary complaint of an eczematous lesion of the left nipple area, shown in Figure 1. The patient had this problem for over a year with fluctuating degrees of severity as the lesion would improve and then worsen. This was the initial visit the patient had regarding this issue and received no prior treatment. She also noticed mild discoloration, pruritis, and drainage of bloody fluid. The patient has had no previous breast-related issues, no trouble with breastfeeding her children, no family history of breast or ovarian cancer, and no prior imaging. There was no palpable mass according to the patient. Bilateral mammography with breast ultrasound was completed which showed a 1 cm mass with multiple irregular calcifications at the 1 o’clock position of the left breast, no axillary adenopathy was noted. Due to the concerning findings, the patient was sent for a needle biopsy. The biopsy of the mass showed ductal carcinoma in situ (DCIS) with a biopsy of a left axillary lymph node being negative for metastatic carcinoma. Estrogen receptor (ER)/progesterone receptor (PR) studies were conducted, and both came back negative. With the leading diagnosis of Paget’s disease of the breast with associated DCIS, the patient was presented with the options of a central lumpectomy versus a mastectomy, both with a sentinel node biopsy. The patient decided on a mastectomy which was performed without complications. The surgical pathology report showed DCIS (high grade), with clear margins, nipple involvement of Paget’s disease of the breast as shown in Figure 2, and sentinel lymph nodes negative for metastases.

## Discussion

Background

Paget disease of the breast (PDB) is an eczematous/ulcerative lesion of the nipple that may spread to the areola and is associated with an underlying malignancy. Approximately 50% of cases are associated with a palpable mass, most commonly being invasive ductal carcinoma [3]. Of the cases with no palpable mass, the underlying malignancy may be invasive or noninvasive. Twelve to 15% of PDB cases are not associated with an underlying malignancy. Over half of the cases of PDB are not estrogen and/or progesterone dependent, however, 84-91% of cases are associated with over-expression of human epidermal growth factor receptor 2 (HER2) [3].

Pathophysiology

The exact cause of PDB is unknown, however, there are two theories as to the origin of this breast cancer. The first theory is the epidermotropic theory which states that neoplastic ductal cells from an underlying malignancy migrate through the ductal system to reach the nipple and areola [4]. Due to the strong association between HER2 over-expression and PDB, there may be a motility factor associated with the HER2 receptor which allows for migration through the ductal system [4]. The epidermotropic theory is the most widely accepted theory for PDB. A second, and much less favorable theory, is the transformation theory which states that PDB is independent of underlying malignancy and arises from epidermal keratinocytes [4]. This theory is less supported due to studies that have expressed the involvement of ducts directly below the nipple in many cases [4].

Presentation and diagnosis

PDB presents as an eczematous lesion of the nipple with associated symptoms such as pruritus, bloody nipple discharge, burning, and bleeding [5]. When diagnosing PDB a thorough history and physical is crucial. Often PDB can be mistaken for a dermatitis or eczema which may delay correct diagnosis of the condition. Recommendations in similar cases with high suspicion of dermatitis or eczema include a short-term, four to six weeks, application of steroids, if the lesion involving the nipple does not resolve, further diagnostic studies for PDB should be pursued [3]. Diagnostic testing for PDB includes mammography, ultrasound, and magnetic resonance imaging (MRI) for detection of underlying mass, needle biopsy of any underlying mass, hormone receptor testing, and full-thickness biopsy of the nipple [3]. Histologic hallmark finding of PDB of the nipple biopsy is Paget cells, which are malignant large cells with clear cytoplasm and prominent nuclei [3].

Treatment

The gold standard treatment for PDB has been mastectomy in the past, however, the increasing popularity of breast-conserving treatment (BCT) in other forms of cancer has introduced the option of BCT along with whole breast radiotherapy for PDB as well. Sentinel lymph node biopsy is paired with either mastectomy or lumpectomy in order to rule out metastasis. While BCT has been researched in various trials for other more common forms of cancer and has presented with a similar recurrence rate to a mastectomy, there is little published data for the use of BCT in cases of PDB. Breast-conserving treatment paired with whole breast radiotherapy provides the ability for breast cancer patients to have a more cosmetically appealing treatment option with retention of breast tissue not associated with malignancy, while still retaining a recurrence rate similar to that of mastectomies. The lack of clinical data could potentially be a cause for skepticism for many patients preventing them from considering BCT with whole breast radiotherapy in their treatment plan.

Prognosis

The prognosis of Paget disease of the breast is dependent on the type of underlying breast cancer. Palpable masses are associated with a poorer prognosis, with a five-year survival range from 20-60% as compared to those with no palpable mass having a five-year survival range from 75-100% [3].

## Conclusions

Paget disease of the breast accounts for only 1-3% of all cases of breast cancer, making it a rather rare presentation. Historically Paget disease of the breast has been treated with mastectomy, however, the increasing prevalence of breast-conserving treatment used in other cancers has posed the question of its efficacy in the case of Paget disease of the breast. With limited clinical data present regarding the reoccurrence rate and efficacy, breast-conserving therapy has yet to become a standard treatment option for Paget disease of the breast.
